# SERS-Active Micro/Nanomachines for Biosensing

**DOI:** 10.3390/bios15020115

**Published:** 2025-02-16

**Authors:** Chenbing Li, Wenqing Zhang, Kai Zheng, Jianhe Guo

**Affiliations:** Guangdong Provincial Key Laboratory of Sensing Technology and Biomedical Instrument, School of Biomedical Engineering, Shenzhen Campus, Sun Yat-sen University, Shenzhen 518107, China; lichb5@mail2.sysu.edu.cn (C.L.); zhangwq63@mail2.sysu.edu.cn (W.Z.); zhengk35@mail2.sysu.edu.cn (K.Z.)

**Keywords:** micro/nanomachines, SERS, localized detection, target capture, biosensing

## Abstract

Surface-enhanced Raman spectroscopy (SERS) has emerged as a powerful noninvasive analytical technique with widespread applications in biochemical analysis and biomedical diagnostics. The need for highly sensitive, reproducible, and efficient detection of biomolecules in complex biological environments has driven significant advancements in SERS-based biosensing platforms. In this context, micro/nanomachines (MNMs) have garnered attention as versatile SERS-active substrates due to their unique structural and motional characteristics at the micro- and nanoscale. This review explores the advantages of integrating MNMs with SERS for biosensing, discussing recent technological advances, various propulsion strategies, and their potential in a range of analytical applications.

## 1. Introduction

Raman spectroscopy, a vibrational spectroscopic technique, holds significant potential for biochemical analysis due to its nondestructive, noninvasive properties and its ability to provide molecular fingerprinting. Compared to fluorometric or colorimetric methods, Raman spectroscopy, based on molecular vibrations, excels in delivering intrinsic molecular information from liquid, solid, and gaseous samples. The narrow emission bandwidth and rich spectral data offered by Raman spectroscopy facilitate the multiplex detection of biomolecules. Furthermore, Raman signals are highly resistant to photobleaching, making them ideal for long-term monitoring under continuous light exposure. However, due to the inherently low efficiency of Raman scattering, detecting analytes at low concentrations remains challenging. This limitation is overcome by surface-enhanced Raman scattering (SERS), which significantly amplifies signal intensity and addresses the issue of weak spontaneous Raman signals [1,2]. With coinage metal nanostructures as SERS substrates, enhancement factors (EF) of 10^6^ to 10^12^ are routinely observed [3]. With the aid of statistical methods, quantitative SERS analysis can achieve an ultralow detection limit far below the single-molecule level [4,5]. Leveraging this high sensitivity, SERS has found widespread application in areas such as pesticide residue detection [6,7], drug assessment [8,9], and hazardous substance analysis [10,11]. In biosensing, SERS is particularly valuable for detecting trace biomolecules that are otherwise difficult to identify using conventional Raman spectroscopy, highlighting its significant potential in biomedical analysis and diagnostics [12,13,14]. Its applications in biomedical fields, such as biomolecular detection [15,16], cancer diagnosis [17,18], pathogen identification [19,20], and cellular imaging [21,22], are particularly noteworthy.

SERS platforms are generally classified into two main categories: colloidal SERS substrates and solid SERS substrates [23,24,25]. Colloidal SERS substrates rely on the aggregation of nanoparticles (NPs) to form hotspots. However, this aggregation process is often chaotic, uncontrollable, and inefficient, leading to reduced reproducibility and homogeneity in SERS detection. In contrast, solid SERS substrates are typically constructed using various fabrication techniques, such as self-assembly, electron beam lithography, and electrochemical methods, to create random or periodic arrays of nanostructures on fixed surfaces. These substrates effectively address the homogeneity issues associated with colloidal platforms. Moreover, solid substrates could also achieve high sensitivity by creating dense and enhanced hotspots generated by arrays of specific nanostructures [26,27]. Despite these advantages, solid substrates often suffer from cleanliness issues, which can decrease detection accuracy after multiple uses. Additionally, the target capture strategy in solid substrates relies on the passive diffusion and adsorption of analytes, which is highly inefficient for detecting trace amounts [28,29]. Thus, the development of new technologies is necessary to address these limitations and enhance both detection efficiency and sensitivity.

Micro/nanomachines (MNMs) represent a novel technology that has the potential to address many challenges faced by traditional SERS platforms. These systems operate at the micro- and nanoscale, converting various energy sources into mechanical motion. MNMs are generally classified into two types based on their energy input: fuel-driven and field-driven [30,31]. Fuel-driven MNMs generate propulsive force through ionic or molecular flows created by chemical reactions, while field-driven MNMs utilize external physical fields, such as acoustic, magnetic, optical, or electric fields, to achieve motion. Due to the different principles governing their propulsion mechanisms, MNMs can be fabricated in various shapes and materials to accommodate specific movement requirements [32,33]. These adaptable structures allow MNMs to serve a wide range of functions, including precision microsurgery, drug delivery, cargo transport, environmental remediation, and medical imaging [34,35]. By modifying surface receptors, MNMs can be tailored to capture and detect different targets, offering significant potential for applications in biomedical sciences.

Micro/nanomachines (MNMs) demonstrate immense potential in the field of biosensing [36,37], which has drawn significant research attention and yielded substantial results. Considerable efforts have been invested in integrating MNMs with conventional analytical detection techniques, enabling sensitive, efficient, rapid, and accurate bioanalysis. For example, the continuous motion of MNMs in solution has been shown to significantly enhance target capture efficiency, resulting in more sensitive and effective detection [38,39,40]. Additionally, MNMs can be directionally controlled to transport biological targets to specific locations, such as fixed sensor surfaces, for precise targeting and detection [41,42]. Furthermore, MNMs exhibit the ability to quickly enter cells due to their strong propulsion capabilities, allowing them to navigate to intracellular targets [43,44]. This characteristic accelerates the internalization of nanomotors into cells, facilitating rapid intracellular sensing [45,46]. Consequently, the unique structural and motional properties of MNMs position them as highly promising tools for advanced biosensing applications.

**Scheme 1:**
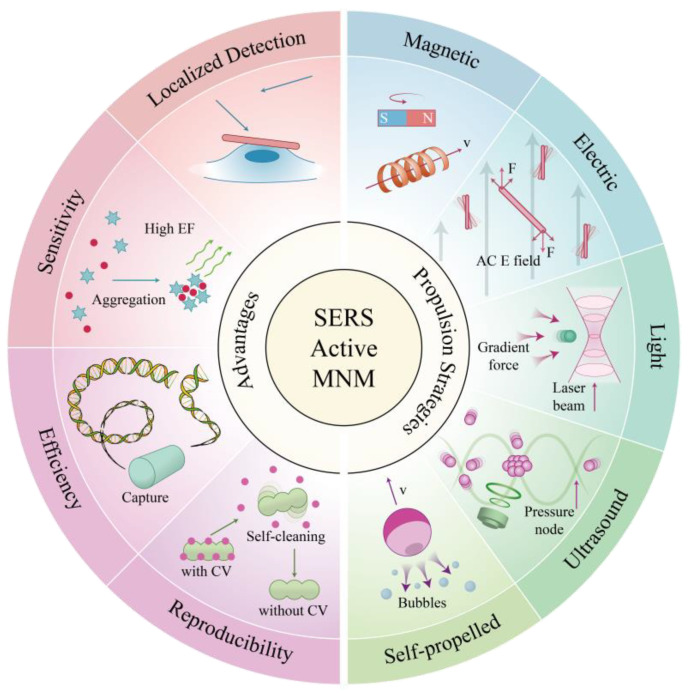
Schematic illustration of the unique advantages and the propulsion strategies of SERS-active MNMs for biosensing applications.

The advantages of MNMs in analyte capture efficiency and directional motion have also garnered significant attention in SERS applications. When combined with SERS-active materials, MNMs can form mobile SERS sensing platforms that effectively enhance detection sensitivity and efficiency, while enabling localized detection and self-cleaning functions. As shown in Scheme 1, this review aims to explore the advantages of SERS-active MNMs, summarize recent developments in this field, and provide insights into their propulsion mechanisms, while highlighting their potential applications across various fields, from medical diagnostics to environmental science.

## 2. Advantage of SERS-Active MNMs

Various SERS-active micro/nanomachines (MNMs) have been developed, each designed to incorporate a plasmonic component essential for their SERS functionality. Typically, there are two primary methods for constructing SERS-active MNMs [47,48]. One method involves directly using plasmonic materials as the core structure of MNMs, such as gold nanotubes. The other method integrates plasmonic materials into MNMs through techniques such as vapor-phase deposition or the in situ growth of plasmonic nanoparticles. Thanks to the versatility of MNMs, these SERS-active systems could support real-time, dynamic sensing and be paired with additional applications, such as drug delivery, targeted drug release, and cellular transport. Compared to conventional SERS substrates, SERS-active MNMs exhibit significant advantages, particularly in localized detection, detection efficiency, sensitivity, and self-cleaning capabilities.

### 2.1. Localized Detection

Localized extracellular detection enables not only the analysis of chemical information on cell membranes but also the evaluation of local physicochemical conditions, which is crucial for understanding physiological processes, disease mechanisms, and therapeutic developments [49]. Considerable research has focused on leveraging MNM manipulation in complex environments to facilitate localized extracellular detection. MNMs can be precisely guided to predetermined locations around cells using external fields, allowing targeted sensing at specific sites. For example, autonomously propelled MNMs have been employed to create microsphere-assisted microscopy, enabling noninvasive “on-the-fly” scanning and high-resolution optical imaging [50]. Moreover, precise manipulation of MNMs has proven useful in localized drug delivery, where nano rockets, for instance, can be directed to specific areas and release drugs in a controlled manner once attached to targeted cells. MNMs also exhibit the ability to deeply penetrate tumor tissue, reaching regions that traditional contrast agents cannot access [51], thereby enhancing tumor imaging and opening new possibilities for the precise treatment of large tumors.

SERS-active MNMs offer significant advantages in localized extracellular detection compared to conventional SERS platforms. Traditional SERS devices often face challenges in locating and contacting hotspots due to the immobilization of hotspots on fixed substrates. In contrast, SERS-active MNMs can be flexibly transported to specific locations, a key step toward efficient targeting and detection. For example, Fan’s group developed a method that uses electrical manipulation and magnetic assembly to position plasmonic nanocapsule SERS sensors for high-sensitivity biochemical detection at precise, predetermined locations, achieving a positional accuracy of 100 nm [52]. Subsequently, they constructed nanotubular SERS-active MNMs capable of analyzing individual cells [53]. Magnetic field-regulated nanotubes were guided to attach to live Chinese hamster ovary cells, where they detected the composition of the cell membrane by SERS spectra. Given that SERS enhancement is distance-dependent, these MNMs selectively capture signals from the cell membrane, avoiding interference from the entire cell.

Thanks to their autonomous propulsion capabilities, MNMs have also been applied in intercellular biological applications. For example, MNMs composed of soft double-micelle microemulsions and bacteria were guided toward targeted cells using external concentration gradients. The bacteria provided propulsion, delivering dye into live cells for the real-time fluorescent imaging of organelles [54]. Additionally, ultrasound-driven nanomotors have demonstrated the ability to enhance cellular interactions with probes and accelerate the internalization process, resulting in more efficient and rapid biomarker detection within cells [44]. In general, intercellular detection using MNMs involves three key steps: first, MNMs are guided to settle around the cell; second, they penetrate the cell membrane; and finally, they perform biosensing within the cell. These approaches can be divided into two categories based on the method of cell entry: MNMs that enter cells via endocytosis and those that reply on strong propulsion to break through the cell membrane.

In recent years, MNMs have been employed for SERS-based intracellular analysis. For example, Liu et al. [55] developed nanomotors with a dual manipulation strategy for intracellular detection. These nanomotors were actively directed to the target cells, where they adhered closely to the cell membrane using a three-dimensional magnetic control platform. The nanomotors then entered the cells via endocytosis, a process enhanced by the oxidative decomposition of glucose due to the GOx-like catalytic activity of Au nanostars on their surface. Large MNMs, unable to enter cells via endocytosis due to the resistance of cell membranes, can penetrate the membranes directly to detect intracellular substances. Chen et al. [56] created a magnetic nanorobot based on carbon nanocoils capable of targeting individual cells with precision and penetrating cell membranes. The helical motion of these nanorobots, combined with strong magnetic propulsion, enabled them to enter cells and obtain SERS signals from cellular plasma and nuclei. Notably, live/dead cell staining assays and animal experiments carried out in this work [56] indicated that these nanorobots based on carbon nanocoils did not cause obvious cell apoptosis (in 24 h) or tissue damage (in 7 days).

### 2.2. Sensitivity

Sensitivity is a critical concern in Raman spectroscopy, with several factors influencing the sensitivity of SERS-active MNMs, including concentration effects (driven by MNM movement and external physical fields), photonic effects (stemming from the 3D structure of MNMs), and hotspot density and enhancement factors (EF) (resulting from controlled aggregation).

The concentration effect of SERS-active MNMs arises from both their dynamic movement and the application of external physical fields. Unlike static sensing strategies, the dynamic movement of MNMs increases the capture probability and the adsorption efficiency, thus increasing the localized analyte concentration within hotspots (the surface of SERS nanoparticles). Han et al. [57] demonstrated this effect by showing that active microengines achieved a Raman signal approximately five times more intense than that of inactive counterparts, highlighting the role of MNM motion in boosting SERS signal intensity. Additionally, external physical fields can be used to aggregate molecules, further increasing analyte concentration. For example, Liu et al. [58] successfully integrated hybrid plasmonic nanocapsules onto photonic crystal slabs (PCSs) using electrical fields. These fields attracted analyte molecules to the nanocapsule surfaces, where high-density plasma hotspots were located, resulting in a 19% to 45% increase in signal intensity. Furthermore, the coupling of guided-mode resonance effects in PCSs with localized surface plasmon resonance (LSPR) contributed to a fivefold increase in detection sensitivity.

Hotspot density and photonic effects, influenced by the 3D structure of MNMs, are crucial factors in improving SERS sensitivity. Xu et al. [53] designed MNMs using hollow silica nanotubes coated uniformly with silver nanoparticles (AgNPs), creating a structure with high hotspot density and a near-field coupling effect between the inner and outer AgNP layers. These SERS-active MNMs achieved an EF as high as 7.2 × 10^9^. Similarly, Guo et al. [59] used diatom frustules as micromotor bodies, featuring ordered arrays of nanopores that functioned as natural photonic crystals. These structures resonated with localized surface plasmons and increased the surface area, effectively amplifying the enhancement factor. In another study, Fan et al. [60] developed hierarchically structured micromotors (HSMs) with two designs: one featuring nanocap arrays and the other nanobowl arrays on their outer walls. Compared to micromotors with smooth walls, the nanocap and nanobowl structures exhibited approximately 3.5 and 2.2 times higher Raman intensity, respectively. These examples demonstrate how MNM structures can be engineered to construct and enhance hotspots, leading to highly sensitive SERS sensing.

The controllable aggregation of MNMs is another effective strategy for enhancing SERS sensitivity, as it facilitates both hotspot construction and molecular enrichment. The aggregation of MNMs can be easily controlled and reversed by applying or removing external fields. Recent studies have explored the use of MNM aggregation to improve SERS detection. Luo et al. [61] developed a highly sensitive, reproducible, and stable active enrichment platform in which two opposing acoustic waves generated periodic pressure nodes. Functionalized gold nanorods migrated toward these nodes, forming stable assemblies within 1–2 s. This mechanism enabled the high-sensitivity SERS detection of COVID-19 biomarkers at concentrations as low as 6.15 × 10^−13^ M in nanoliter (10^−7^ L) samples. When the ultrasound was turned off, the nanorods redispersed, and no significant Raman signals were detected. Similarly, Asunción-Nadal et al. [62] developed photophoretic Au@MoS_2_ micromotors powered by the same laser source used for Raman spectroscopy, eliminating the need for an additional laser. These micromotors aggregated at the laser focus, and due to the long-range photophoretic effect, a large number of micromotors were aggregated. These aggregated micromotors exhibited a SERS signal that was 15 to 18 times stronger than that of decentralized micromotors. This universal, label-free technique offers the potential for the highly selective detection of a wide variety of analytes.

### 2.3. Efficiency

In SERS biosensing, the efficiency of detection is highly dependent on the successful interaction between sensors and analytes, due to surface plasmonic resonance. Molecule capture on biosensor surfaces involves three key processes: analyte convection driven by flow, diffusion toward the sensor surface, and absorption onto the surface. As suggested by the Nernst diffusion theory, there is a layer of static liquid between the solid surface and mobile liquid. Inside the so-called Nernst diffusion layer, the transport of molecules only occurs in the form of passive diffusion driven by the concentration gradient. In dilute analyte solutions, molecules must traverse a diffusion layer of several micrometers, reaching the sensor surface via a slow diffusion process. As a result, detection efficiency is largely limited by this slow molecular diffusion, particularly in cases where the concentration gradient is low.

Unlike conventional biosensors, MNM-based sensors can actively move, effectively stirring and mixing the surrounding fluid to facilitate rapid contact with analytes. The mechanical movement of MNMs enhances molecular capture, making them a compelling addition to biosensing strategies. When operating in microvolume samples, MNMs function like tiny stirrers, accelerating the mixing of trace target analytes [63]. For example, a micromotor can achieve a homogeneous dye mixture within two minutes, whereas static conditions would require up to ten minutes [64]. Furthermore, bubble-propelled MNMs, which generate tail microbubbles, have been shown to further accelerate mixing and improve detection efficiency [42,65].

Zhang et al. [66] developed MNM-based biosensors for detecting C. diff toxins by integrating fluorescent carbon nanodots with magnetic spores. These active MNM biosensors achieved detection within ten minutes, demonstrating significantly higher efficiency compared to static biosensors. Similar improvements in efficiency due to MNM motion have been observed in SERS detection. For instance, when measuring the same concentration of crystal violet (CV) solution, magnetically actuated hydrogel-based planar microswimmers (MHMs) produced a Raman signal 170% stronger than that of inactive probes [67]. The increased capture of CV molecules in the same time frame highlights the higher efficiency of active MHM sensors.

Mechanical rotation is another effective method for accelerating analyte capture and improving detection efficiency. By reducing the thickness of the diffusion layer around nanosensors, mechanical rotation enhances diffusion and capture efficiency. Notably, the diffusion layer thinning effect resulted by the surrounding continuous flows cannot be achieved in transient state during Brownian motions. Guo et al. [59] synthesized a rotary micromotor-sensor system designed to accelerate analyte capture through high-speed rotation. This approach increased the efficiency of DNA molecule capture by at least four times. A micromotor-sensor rotating at 1200 rpm reached 95% of DNA capture equilibrium within approximately 7 min, compared to 28 min for a static microsensor.

### 2.4. Reproducibility with Self-Cleaning

SERS is a distance-dependent phenomenon that enhances molecular signals by capturing analyte molecules on the sensor surface. The interactions between the sample and SERS substrates, such as van der Waals forces and electrostatic binding, often cause analyte molecules to remain on the sensor surface after initial detection. These residual molecules can significantly impact the accuracy of subsequent measurements, ultimately compromising the reproducibility of SERS tests.

SERS-active MNMs overcome this limitation through their dynamic self-cleaning capability. Numerous studies have demonstrated that MNMs are capable of delivering and releasing molecules, highlighting their potential for self-cleaning. For instance, Xu et al. showed that the rotational movement of MNMs can accelerate the release of molecules from their surfaces [68]. The rotation reduces the thickness of the stationary diffusion layer and, due to the significantly lower analyte concentration in the bulk solution compared to the nanomotor surface, molecules are quickly removed from the MNM surface. This mechanical rotation effectively enables self-cleaning, preventing residual molecules from affecting subsequent measurements.

Wang et al. [69] demonstrated a self-cleaning strategy using the self-rotation of a magnetic nanomotor-based SERS probe (MNM-SP). After detecting crystal violet (CV) with the MNM-SP, the probe was immersed in deionized (DI) water, yet still exhibited a clear Raman signal due to CV residue. However, over the course of a 30-min rotation, the CV signal gradually diminished to an undetectable level. When the probe was subsequently placed in a rhodamine 6G (R6G) solution, distinct R6G signals were detected, confirming that self-rotation effectively removed residual analytes, allowing the SERS probe to be recycled for future use.

## 3. Different Propulsion Strategies and Applications of SERS-Active MNMs

### 3.1. Self-Propelled SERS-Active MNMs

Self-propelled MNMs are micro/nanoscale machines that convert chemical or biochemical energy into kinetic energy. These propulsion mechanisms can be broadly classified into three types: bubble-driven, self-electrophoresis-driven, and self-diffusion-driven systems [35,70].

(1) Bubble-driven MNMs utilize asymmetric catalytic structures that generate bubbles through redox reactions of fuels in solution. The bubbles are ejected from the surface of the MNMs, causing momentum in the opposite direction, which propels the MNMs.
(2) Self-electrophoresis-driven MNMs are constructed using two materials with different electric potentials. Oxidation and reduction reactions occur in distinct regions of the MNM, creating a localized electric field due to the asymmetric distribution of ionic products. This induces electrophoretic motion.
(3) Self-diffusion-driven MNMs rely on a concentration gradient of reaction product created by the asymmetric distribution of catalytic materials. The diffusion of reaction products along this gradient propels the MNMs forward.

Self-propelled MNMs offer distinct advantages, such as low cost and high propulsion speed, as they utilize chemical fuels in solution and do not require external devices for propulsion. However, they also have significant limitations. The catalytic reactions driving their motion are often uncontrollable, leading to a lack of precise directional control. Additionally, their movement is dependent on specific fuels, restricting their use to environments where these fuels are present. As a result, controlled motion is difficult to achieve, and these MNMs often need to be combined with other physical fields for directional guidance. Despite this, their disordered movement can facilitate the enrichment of analytes in solution, making them effective in molecular detection applications.

**Figure 1:**
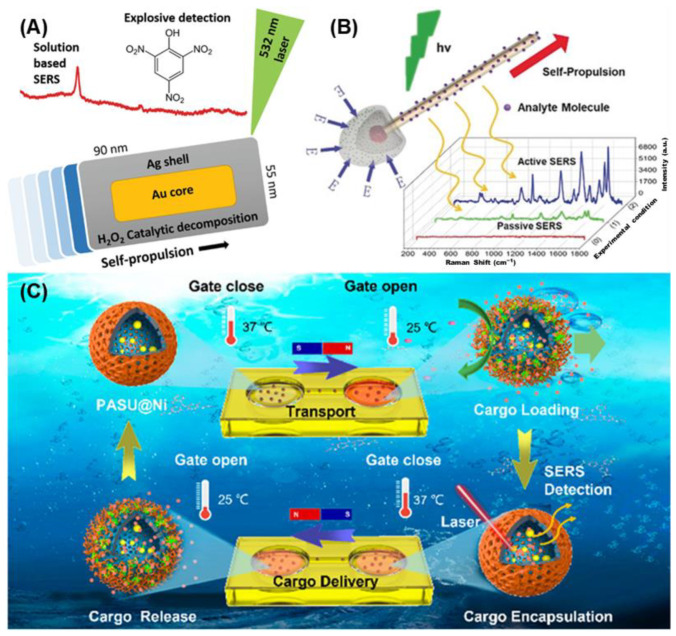
Self-propelled SERS-active MNMs: (**A**) Schematic illustration of self-propulsion gold/silver nanomotors serving as mobile SERS sensors [71]. Copyright © 2019 WILEY-VCH. (**B**) Schematic illustration of photocatalysis-powered matchlike nanomotors for light-guided SERS sensing [72]. Copyright © 2018 WILEY-VCH. (**C**) Schematic illustration of the SERS-active MNM and its controllable targeting, temperature-responsive loading, encapsulation, and release [73]. Copyright © 2022 American Chemical Society.

Au and Ag are ideal materials for integrating SERS with self-propelled MNMs, as they are commonly used in both SERS applications and the construction of self-propelled MNMs. Pumera et al. [71] synthesized self-propelled MNMs composed of gold nanorods with silver shells using wet chemical methods (Figure 1A). These MNMs were able to propel themselves in low concentrations of hydrogen peroxide and detect trace amounts of picric acid, demonstrating their effectiveness as mobile SERS platforms. Furthermore, by replacing CTAB with MTAB on the surface, the MNMs achieved a lower detection limit (0.1 μM) for picric acid while also improving biocompatibility. This highlights the importance of avoiding CTAB in biomedical applications to enhance safety and compatibility.

Photocatalysis is another promising mechanism for driving self-propelled MNMs, offering excellent biocompatibility. Ma et al. [72] developed photocatalysis-powered MNMs resembling matchsticks for light-guided SERS sensing (Figure 1B). These MNMs were composed of SiO_2_-coated silver nanowires with an AgCl tail. Upon exposure to UV light, AgCl undergoes photocatalytic decomposition, producing protons (H^+^) and chloride ions (Cl^−^). The difference in diffusion speeds creates an electric field, generating a force that propels the MNMs toward the light source, demonstrating positive phototaxis. As the MNMs aggregate under UV light, they enhance the local Raman signals, enabling the detection of specific cells and amplifying their Raman signatures.

A notable application of enzymatic SERS-active MNMs has also been demonstrated in molecule delivery and detection (Figure 1C) [73]. These MNMs encapsulated gold nanodots as plasmonic probes within hollow mesoporous silica nanospheres. The surface of the MNMs was coated with urease for chemical propulsion and nickel layers for magnetic propulsion. Additionally, temperature-responsive polymers on the surface allowed for control over the nano-through-band, enabling molecular cargos to be loaded and released based on environmental temperature changes. The active motion of these MNMs significantly accelerated the enrichment of molecules in solution, with the amount of loaded cargo detected in real-time using SERS.

Ma et al. [74] later advanced the design of enzymatic SERS-active MNMs for capturing and sensing exosomes. These urease-powered MNMs were selectively modified with amino groups to control enzyme distribution. The micromotors were also coated with gold for SERS detection and functionalized with aptamers via Au-S bonds for exosome trapping. Both experimental results and simulations showed that these micromotors, with internally modified urease, exhibited faster movement speeds. The catalytic reaction was primarily confined to the internal cavity of the micromotors, concentrating the fluidity at the tail, thereby minimizing interference with exosome binding on the aptamer-coated surface. This design significantly enhanced both exosome capture efficiency and detection sensitivity.

**Figure 2:**
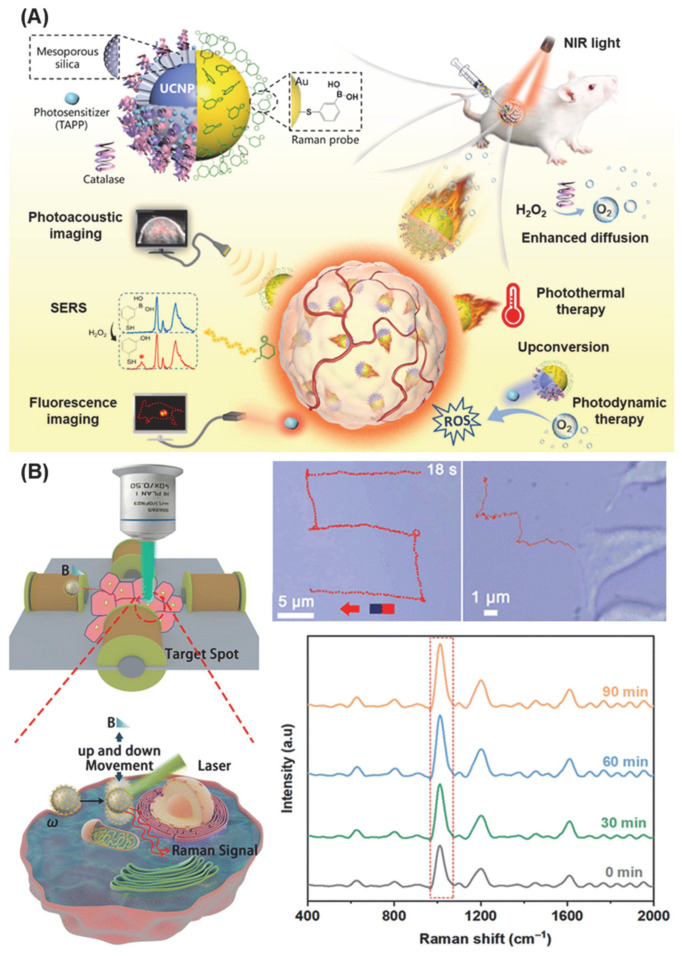
Self-propelled SERS-active MNMs: (**A**) Schematic illustration of the dual-source powered MNMs for SERS biosensing and multimodal cancer phototheranostics [75]. Copyright © 2022 Elsevier Ltd. (**B**) Schematic illustration, motion snapshots and Raman spectra of targeted cellular SERS sensing by the nanoenzymatic MNMs [55]. Copyright © 2023 Royal Society of Chemistry.

Recently, Ma et al. [75] further developed enzyme-propelled MNMs with integrated functions for cancer phototheranostics (Figure 2A). These innovative MNMs combine multiple diagnostic and therapeutic modalities, including SERS sensing, fluorescence imaging, photoacoustic imaging, photodynamic therapy, and photothermal therapy. Gold and Raman probe molecules were deposited on one side of the MNMs, enabling the specific SERS-based detection of hydrogen peroxide, allowing for both in vitro and in vivo quantitative analysis in the tumor microenvironment. On the opposite side, hydrogen peroxidase and photosensitizers were deposited to enable catalytic decomposition and photothermal activity, driving the nanomotors in low concentrations of H_2_O_2_. These multifunctional MNMs hold great promise for the intelligent detection, diagnosis, and photothermal treatment of tumors.

Liu et al. [55] similarly developed nanoenzymatic MNMs that integrate both diagnostic and therapeutic functions (Figure 2B). The MNMs were surface-modified with gold nanostars (AuNS), which catalyze glucose reactions to promote their diffusion. These MNMs, with Fe_3_O_4_ nanoparticles as their core, demonstrated directional movement toward target cells and oscillatory motion. The localized surface plasmon resonance effect of the AuNS enabled both photothermal therapy and SERS detection. These MNMs were able to detect crystal violet with a detection limit of 10^−8^ M, allowing for the effective collection of Raman information from low-concentration substances within cells.

In summary, self-propelled MNMs offer significant advantages in terms of propulsion speed, making them highly effective for analyte enrichment and improving detection efficiency. Their ability to utilize natural fuels, such as urea or hydrogen peroxide, found in physiological environments like normal tissues or tumor microenvironments, enhances their biocompatibility. However, the inability to precisely control the catalytic reaction sites on their surfaces remains a challenge, making it necessary to combine them with other physical fields for controlled motion and directional accuracy.

### 3.2. Magnetically Propelled SERS-Active MNMs

Magnetic fields are extensively used to manipulate MNMs due to their ability to provide precise control and well-defined propulsion mechanisms. Paramagnetic or ferromagnetic MNMs in magnetic fields can be aligned, rotated, transmitted, or dragged, driven either by a magnetic field gradient or magnetic torque [76]. In gradient-driven MNMs, the force applied is determined by the induced magnetic moment of the MNMs and the gradient of the magnetic field. This interaction causes the MNMs to move toward the region of the strongest gradient in a nonuniform magnetic field. One of the advantages of this method is that it does not require specific design modifications, making it versatile in spatially varying magnetic fields. On the other hand, torque-driven MNMs possess magnetic moments that align with the direction of the magnetic flux density. The alignment generates a torque, which rotates the MNMs until they are parallel to the magnetic field. By applying different types of magnetic fields, such as rotating, oscillating, or pulsed fields, and adjusting the magnetic flux density in real time, precise control of MNM movement can be achieved. This allows for sophisticated manipulation techniques, enabling high-speed rotation and precise positioning. Such control makes magnetically propelled MNMs highly effective in functions such as analyte enrichment, self-cleaning, and sensitive SERS detection, all of which benefit from their ability to form localized hotspots.

**Figure 3:**
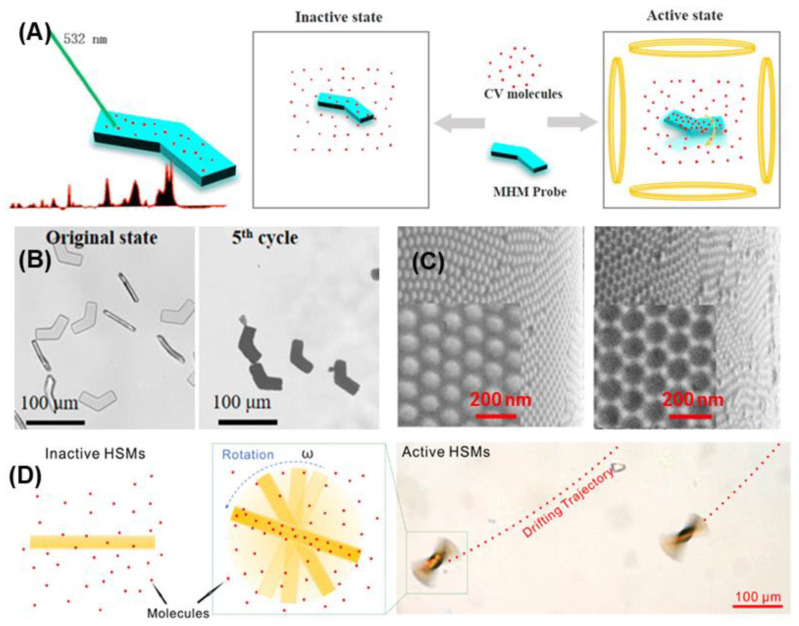
Magnetically propelled SERS-active MNMs: (**A**,**B**) Schematic illustration of the SERS enhancing principle and optical images of magnetically actuated hydrogel-based achiral planar microswimmers [67]. Copyright © 2023 Frontiers Media. (**C**,**D**) SEM images of Au nanostructure arrays on the tubular MNMs and schematic illustration of the SERS enhancement effect in accelerating the molecular adsorption [60]. Copyright © 2020 American Chemical Society.

U. Cheang et al. [67] developed magnetically actuated hydrogel-based MNMs and demonstrated their ability to enrich analytes for SERS detection (Figure 3A,B). The MNMs were fabricated using photocured hydrogel, with Fe_3_O_4_ nanoparticles for magnetic actuation and Ag nanoparticles for SERS detection uniformly distributed on their surface. By controlling a rotating magnetic field, these MNMs achieved high-speed rotation with excellent swimming efficiency. Even at a low rotational speed of 10 Hz, the microswimmers significantly improved detection efficiency, reaching a detection limit of 10^−8^ M for crystal violet.

T. Qiu et al. [60] improved the SERS performance of magnetically driven MNMs by creating hierarchical structures (Figure 3C,D). Using nanoimprint and rolling origami techniques, they fabricated Au nanostructure arrays on the outer walls of tubular MNMs, generating high-density plasmonic hotspots. When subjected to a rotating magnetic field, these MNMs exhibited a significant increase in SERS intensity, largely due to active molecular enrichment during rotation. The detection limit for Rhodamine 6G on the rotating MNMs was over 20 times lower than that of inactive MNMs.

**Figure 4:**
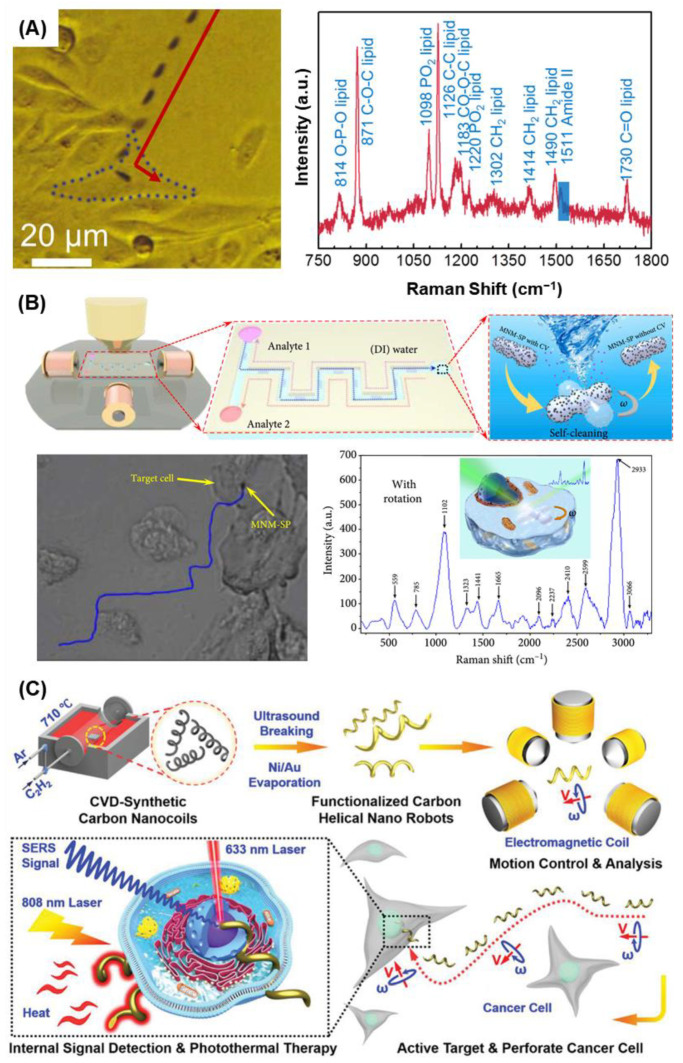
Magnetically propelled SERS-active MNMs: (**A**) Localized detection by SERS-active MNMs for specific cell analysis [53]. Copyright © 2013 WILEY-VCH. (**B**) Motion control, SERS sensing, and self-cleaning of SERS-active MNMs for intracellular biosensing [69]. Copyright © 2020 American Association for the Advancement of Science. (**C**) Schematic illustration for the fabrication of carbon helical structure-based SERS-active MNMs and the application of targeted cell penetration for SERS sensing and photothermal cancer therapy [56]. Copyright © 2022 WILEY-VCH.

D. Fan et al. [53] were the first to demonstrate the use of magnetically driven SERS-active MNMs for specific cell analysis. They designed rod-shaped bifunctional plasmonic-magnetic MNMs with embedded solid magnets and dual-surface-coated Ag nanoparticles. Controlled by a gradient magnetic field, these MNMs exhibited precise speed and direction control. The uniform coating of Ag nanoparticles on both the inner and outer surfaces of the tubular MNMs resulted in uniform enhancement factors (EFs) as high as 10^9^. The MNMs successfully performed the localized detection of individual CHO cells (Figure 4A), providing detailed spectral information about the cell membrane components.

X. Ma et al. [69] extended the application of SERS-active MNMs to intracellular biosensing (Figure 4B). To facilitate cell entry, the MNMs were designed with dimensions smaller than 1 micrometer and were self-assembled using magnetic nanoparticles, coated with a thin layer of silica and Ag nanoparticles. Under a gradient magnetic field, these MNMs could be precisely navigated to target positions for SERS detection. Applying a rotating magnetic field further enhanced analyte enrichment and enabled self-cleaning in DI water after detection. The MNMs were guided into cells via endocytosis, where they provided significantly more detailed information on intracellular components compared to static MNMs.

Recently, X. Ma et al. [56] developed SERS-active MNMs for intracellular biosensing, which also incorporated photothermal therapy functions. These MNMs were composed of carbon helical structures coated with magnetic and gold layers (Figure 4C). The rotating MNMs could mechanically perforate cell membranes and precisely target intracellular sites. In addition to their excellent SERS detection capabilities, these MNMs acted as photothermal therapy platforms, effectively killing cancer cells by leveraging the photothermal properties of carbon and gold.

In summary, magnetic field-driven MNMs are widely used due to their ease of operation and well-established principles. Their excellent biocompatibility, especially with low-frequency magnetic fields, makes them ideal for biosensing applications. However, precise magnetic field control requires expensive and bulky equipment, which may limit their applicability in certain environments.

### 3.3. Electrically Propelled SERS-Active MNMs

Electric field propulsion is also one of the mainstream techniques for manipulating MNMs due to its lower cost and commercial viability compared to magnetic field systems. These systems rely on electrodes and commercial power supplies, making them more accessible for widespread use. Unlike magnetic field propulsion, which requires specific materials, electric fields can drive a broader range of nanoobjects, including metallic particles, droplets, and microbubbles, all of which respond to the AC/DC fields generated by microelectrodes. Common propulsion mechanisms include electrophoresis, dielectrophoresis, electrowetting, and electroosmosis [77,78,79,80]. Electric tweezers, composed of a quadripolar microelectrode, are frequently used to achieve two-dimensional manipulation [79]. This enables MNMs to perform precise linear motion, high-speed rotation, and alignment under different electric field conditions. The ability to precisely control motion makes electric-field-propelled MNMs particularly promising for in vitro biomedical applications, where they can localize the detection sites and perform self-cleaning functions during SERS-based detection.

**Figure 5:**
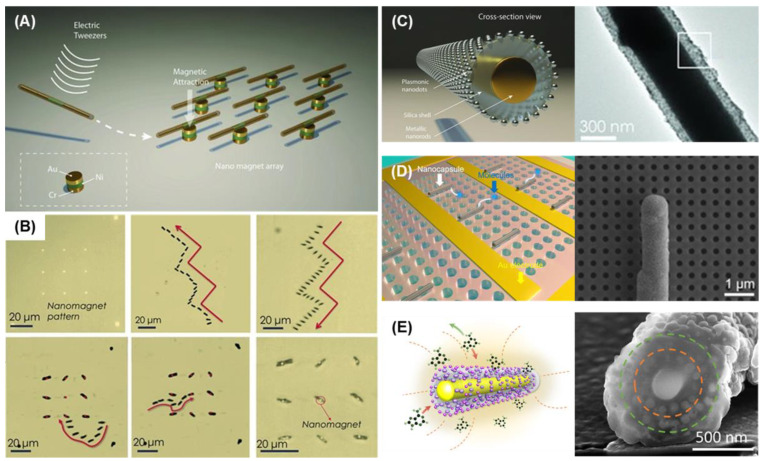
Electrically propelled SERS-active MNMs: (**A**,**B**) Assembly of the SERS-active rotary MNMs array by the electric tweezers [81]. Copyright © 2012 WILEY-VCH. (**C**) Schematic illustration and SEM image of a SERS-active rotor [81]. Copyright © 2012 WILEY-VCH. (**D**) Schematic illustration and SEM image of nanocapsule MNMs on the crystal plates [58]. Copyright © 2017 American Chemical Society. (**E**) Schematic illustration and SEM image of the superstructural nanosensors [84]. Copyright © 2018 American Chemical Society.

D. Fan et al. [52,81] were the first to combine electrically propelled MNMs with SERS, demonstrating highly precise localized detection (Figure 5A). The MNMs were equipped with a SERS-active rotor and magnetic bearing, where magnetic components allowed the rotor to be flexibly assembled via magnetic attraction [52,82]. As shown in Figure 5B, driven by the electrophoretic motion, the SERS rotor could be transported to specific target locations with a positional accuracy of 100 nm [78]. The magnetic bearing stabilized the rotor during rotation, minimizing drift and enhancing the stability and reliability of SERS detection.

A nanocapsule structure was designed by X. Xu et al. for the SERS-active rotor with significantly enhanced capabilities (Figure 5C). These nanocapsules featured nanorods coated with silica and silver nanoparticles. Silica shells, synthesized via TEOS hydrolysis, provided growth sites for plasmonic silver nanoparticles, yielding hotspot densities up to 1200/μm^2^ and EF up to 1.1 × 10^11^, enabling single-molecule detection [81].

Under electric tweezers, K. Kim and J. Guo et al. demonstrated that these SERS-active rotors could rotate at a speed of at least 18,000 rpm, and continuously up to 1.1 million cycles [52,83]. X. Xu et al. proved that the rotational movement of MNMs can accelerate the release of molecules from their surfaces [68]. The excellent mechanical and plasmonic properties facilitate the controllable drug release by rotary MNMs and enable real-time monitoring of drug release rates using SERS.

C. Liu et al. [58] developed an integrated system combining two enhancement mechanisms to boost SERS sensitivity and efficiency by electrically propelled SERS-active MNMs (Figure 5D). The system featured plasmonic nanocapsules as MNMs, lithographically patterned photonic crystals as substrates, and interdigital microelectrodes for electrical manipulations. Electric fields applied to the microelectrodes facilitated the assembly of nanocapsule MNMs on the crystal plates and generated electrokinetic flows to aggregate analytes on nanocapsule hotspots. Through the combined effects of localized surface plasmon resonance (LSPR) and electrokinetic molecular focusing, the system achieved EF up to 2 × 10^9^, enabling single-molecule sensitivity.

J. Liu et al. [84] also developed porous superstructural nanosensors as SERS-active MNMs (Figure 5E). By alternately coating gold nanorods with silica shells and polystyrene nanoparticles templates, silica shells with numerous nanocavities were created, serving as growth sites for silver nanoparticles. The porous structure prevented plasma quenching of the silver nanoparticles and offered a significantly larger surface area compared to planar designs, increasing hotspot density. These nanosensors allowed for the precise transport by electrophoresis and the voltage-controlled release of attached molecules, enabling the real-time in situ detection of controllable release via SERS.

While many electric field propulsion mechanisms remain underexplored, they are widely used in various applications. The high-speed rotation of robotic SERS sensors under a high-frequency AC field offers distinct advantages for monitoring drug release via SERS. Additionally, the simplicity and low cost of electric field systems make them easy to operate and commercially viable. However, one major limitation is their low biocompatibility, making them less suitable for in vivo applications.

### 3.4. Light-Propelled SERS-Active MNMs

Light, being one of the most readily accessible energy sources, usually captures and propels MNMs by the field gradients of light, such as single-beam gradient force traps, plasmonic tweezers, and photothermal gradient fields. In single-beam gradient force traps, also known as optical tweezers, the convergence of light beams generates an electric field intensity gradient, with the highest intensity at the point of convergence [85]. Micro- and nanoscale MNMs are polarized by the electric field and move toward regions of higher field strength. However, conventional optical tweezers are limited in their ability to control microscale MNMs due to weak interactions between light and these small particles. Plasmonic tweezers, which couple the electromagnetic field’s electric component with the resonant oscillations of conduction electrons in metal MNMs, solve this issue by enabling direct control over their motion [86]. This trapping effect allows MNMs to be suspended in a specific position for extended periods, making them ideal for long-term SERS detection and monitoring. Light-induced photothermal propulsion is a different strategy based on the photothermal effect, which occurs when asymmetrically structured MNMs create temperature gradients under light irradiation [87].

**Figure 6:**
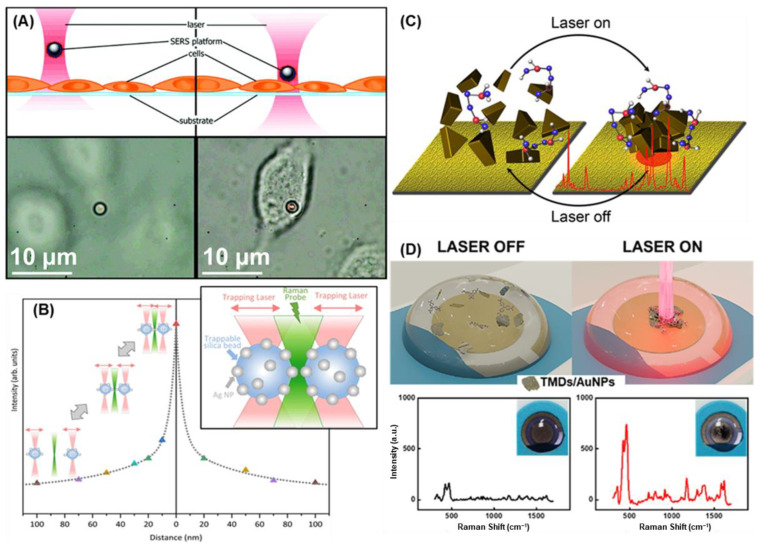
Light-propelled SERS-active MNMs: (**A**) Precise single cell localization and SERS sensing by MNMs controlled by optical tweezers [88]. Copyright © 2015 Royal Society of Chemistry. (**B**) Different SERS enhancement effects achieved by adjusting the distance between MNMs trapped by optical tweezers [89]. Copyright © 2021 Springer Nature Limited. (**C**) Schematic illustration of dynamic manipulation of gold nanotriangle-based MNMs assembled on the plasmonic substrate [90]. Copyright © 2016 American Chemical Society. (**D**) Schematic illustration of the dynamic SERS sensing by Au@MoS_2_ MNMs under optical tweezers and the SERS enhancement effect [62]. Copyright © 2023 American Chemical Society.

Stetciura et al. [88] first used optical tweezers to navigate the SERS-active MNMs to a certain cellular compartment and detect the intracellular composition following cellular uptake (Figure 6A). The SERS-active MNMs were constructed by decorating gold nanoparticles on silica microspheres. They then employed a continuous-wave infrared diode laser to form a light-trap capable of capturing these MNMs. Under laser guidance, the MNMs approached the target cellular surface, and when the laser was turned off, the MNMs adhered to the cell surface. The MNMs were later engulfed by the cell, enabling SERS detection. This technique holds great promise for localized intracellular composition and microenvironment analysis. Similarly, Dai et al. [89] demonstrated an optical-tweezers-controlled hotspot by capturing two silver nanoparticle-coated MNMs (Figure 6B). When the gap between the two MNMs was narrowed by the dual-trap optical tweezers, SERS hotspots were formed, allowing for the detection of proteins in a microfluidic flow chamber and enabling persistent single-molecule measurements. This approach efficiently adjusts hotspots, generating tunable and reproducible SERS enhancement.

In addition to laser traps, light can be employed to assemble plasmonic nanoparticles through the photothermal effect. Zheng et al. [90] utilized a laser to create a temperature-gradient field via the thermophoresis induced by the photothermal effect, which led to the rapid formation of gold nanotriangle (AuNT) assemblies at the laser spot (Figure 6C). This approach allows for the flexible and fast assembly of SERS hotspots. Furthermore, Escarpa et al. [62] developed a type of Au@MoS_2_ MNMs driven by laser for SERS detection (Figure 6D). Upon light irradiation, the MoS_2_ semiconductor generates electron–hole pairs and increases lattice temperature, leading to the aggregation of MNMs through photothermal-induced motion. The MNMs could harvest the target analytes during the accumulation, which lead to increases in both the density of SERS-active MNMs and, concomitantly, the analyte concentration. Notably, the same laser used for Raman detection also serves as the light source for the optical tweezers, eliminating the need for an external motion source and significantly simplifying the experimental setup. The combined effects of the optical tweezers and the localized surface plasmon resonance (LSPR) of AuNPs greatly enhance Raman detection efficiency.

Thanks to their unique propulsion mechanism, light-propelled SERS-active MNMs can efficiently aggregate analytes and enable localized SERS detection, making them highly promising for various SERS applications. However, these MNMs also face certain limitations. Their relatively slow motion and limited motion versatility restrict their ability to handle more complex tasks, such as cargo transport and self-cleaning, which in turn limits their broader applicability.

### 3.5. Ultrasound-Propelled SERS-Active MNMs

Ultrasonic propulsion is a highly promising technique for MNM propulsion due to its biocompatibility and accessibility. Ultrasound is generated using piezoelectric materials, which, when subjected to an alternating current, vibrate at ultra-high frequencies due to the inverse piezoelectric effect. Quartz, with its excellent properties, is often the material of choice for ultrasound generation. Depending on the cutting type (AT or ST cutting), quartz crystals vibrate in different directions, producing either body acoustic waves (BAW) or surface acoustic waves (SAW) [91,92]. Single beam acoustic tweezers based on BAW operate similarly to optical tweezers, trapping particles through the gradient of acoustic forces. In contrast, SAW works differently; as SAW propagates through a liquid medium and interacts with a fluid, the leakage waves create acoustic radiation forces. In standing wave acoustic tweezers, these forces trap objects at pressure nodes or wave bellies based on their physical properties. Changes in acoustic wavelength allow for the precise control of MNM movement by manipulating the position of pressure nodes or wave bellies. This trapping effect provides significant advantages in analyte and SERS-active MNM enrichment, making ultrasonic propulsion highly suitable for SERS applications.

**Figure 7:**
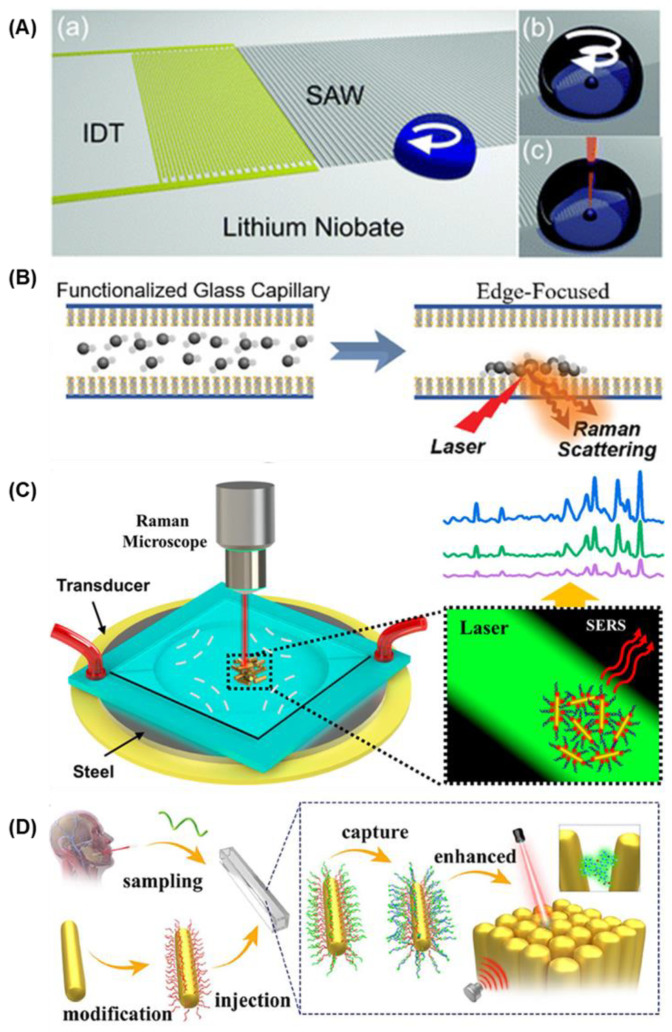
Ultrasound-propelled SERS-active MNMs: (**A**) Schematic illustration of (a) interaction of a surface acoustic waves with a droplet and (b,c) aggregation process of SERS-active nanoparticle [93]. Copyright © 2013 Royal Society of Chemistry. (**B**) Schematic illustration of concentrating targets by MNMs using SAWs [94]. Copyright © 2020 WILEY-VCH. (**C**) Schematic illustration of the aggregation of SERS-active MNMs with an acoustic field and the enhancement of Raman signals [97]. Copyright © 2020 American Chemical Society. (**D**) Schematic illustration of gold nanorod-based MNMs actively capturing the target fragment and assembling by acoustic field for SERS detection [61]. Copyright © 2023 American Chemical Society.

Cooper et al. [93] first successfully employed SAW generated by a single interdigitated transducer to aggregate SERS-active nanoparticles in microdroplets, lowering the detection limit for SERS (Figure 7A). The microdroplet broke the symmetry of the SAW interaction, which induced a circular flow in the microdroplets. The aggregation of SERS-active nanoparticles enabled SERS analysis of the oxidative damage in cells. However, this method is limited in the droplet analysis, and the precise positioning of the droplets is required.

The trapping effect of standing wave acoustic tweezers generated by a pair or a quadripolar of interdigitated transducers enables the enrichment at specific pressure points in bulk solutions. Huang et al. [94,95] demonstrated that a standing wave generated by SAW could enrich MNMs on Ag-ZnO nanorods-based SERS-active substrates (Figure 7B). Analytes bound to the aptamer-conjugated MNMs could also be focused during this process, facilitating the SERS detection of exosomes, DNA, and *E. coli*.

Instead of enriching the analysts, Xu et al. [96,97] demonstrated that by adjusting ultrasound frequency, they could manipulate the aggregation and assembly of gold nanorod-based SERS-active MNMs within an acoustic field. This approach enabled SERS detection at specific locations with a microliter scale and ultra-trace sensitivity, significantly improving detection efficiency and sensitivity (Figure 7C). Xu et al. [61] advanced this technology by developing an integrated SERS detection system for the nucleic acid detection of COVID-19 (Figure 7D). Using standing wave acoustic tweezers, aptamer-modified SERS-active MNMs rapidly aggregated at pressure nodes, allowing for the highly sensitive detection of COVID-19 biomarkers without PCR, achieving a detection limit as low as 6.15 × 10^−13^ M.

While ultrasonic propulsion has fewer applications for MNMs due to limitations in directional control, its efficient aggregation effect holds great promise for SERS applications. Additionally, the excellent penetration and biocompatibility of ultrasound make it a highly favorable method for potential in vivo applications.

## 4. Conclusions and Future Perspectives

Significant progress has been made in the integration of surface-enhanced Raman scattering (SERS) and micro/nanomachines (MNMs) due to extensive research efforts. This review has explored the concepts, advantages, propulsion mechanisms, and various applications of SERS-active MNMs. Compared to conventional SERS substrates, SERS-active MNMs offer distinct advantages, including enhanced localized detection, improved detection efficiency and sensitivity, and self-cleaning capabilities. While initial research primarily focused on demonstrating the feasibility of combining SERS and MNMs, recent studies have increasingly emphasized their potential for in vivo biosensing applications.

Despite these advances, several challenges must be addressed before SERS-active MNMs can be fully integrated into practical biomedical applications. First, biocompatibility remains a critical hurdle. To function as SERS-based biosensing platforms in vivo, both the materials used and the energy sources employed must be biocompatible. For instance, while magnetic fields show high biocompatibility compared to other propulsion methods, many magnetic materials contain toxic metals that can harm living cells. Similarly, silver nanoparticles, which are commonly used for SERS detection due to their high sensitivity, face challenges related to chemical stability and biological toxicity. Developing alternative materials, such as gold-coated nanoparticles or biocompatible polymers, could provide a viable solution to these challenges.

Second, the reliability and reproducibility of SERS-active MNMs are critical factors that need to be improved. The fabrication of these systems often results in high variability between and within batches, which can affect the consistency of SERS detection. Additionally, the complex biological environments in which these devices operate make it difficult to achieve consistent signals. Achieving high reproducibility in SERS signals will require advances in fabrication techniques that can ensure uniform hotspot distribution and consistent signal enhancement.

Third, detecting multiple biomolecules within cells and tissues remains a significant challenge due to the interference caused by the intrinsic biological signals from complex biological matrices. Traditional data processing methods are often inadequate for analyzing the intricate SERS spectra generated in these environments. However, the integration of machine learning and deep learning offers new opportunities. For example, Principal Component Analysis (PCA), an unsupervised machine-learning algorithm widely used in SERS analysis, could simplify the data visualization and pattern recognition by reducing the dimensionality of the spectra data. AI algorithms can also significantly enhance the accuracy of SERS detection by improving noise reduction, enabling the more precise identification of multiple analytes in complex mixtures. Furthermore, deep learning models can automate pattern recognition in SERS spectra, leading to faster and more reliable diagnostics. Future advancements in AI-driven data processing could revolutionize the way SERS-active MNMs analyze and interpret complex biological samples, enabling real-time, multicomponent analysis.

As discussed earlier, the various propulsion mechanisms—such as self-propelled, magnetic, electric, light, and ultrasonic propulsion—offer unique advantages for different applications. Self-propelled MNMs show potential in autonomous systems where external control is limited, while physical fields offer more precise control, which is essential for targeted biosensing. Future research should focus on optimizing these propulsion systems for specific applications, such as high-precision diagnostics, drug delivery, and in vivo medical imaging. Beyond biomedical applications, SERS-active MNMs hold promise in a variety of other fields. For instance, they can be used in environmental monitoring to detect trace levels of pollutants, offering the highly sensitive, in situ analysis of environmental toxins. In food safety, SERS-active MNMs could enable the rapid detection of contaminants, ensuring safer and more reliable food supply chains. Furthermore, the integration of MNMs with SERS platforms opens up new possibilities for industrial applications, such as process monitoring and quality control, where precise and efficient chemical analysis is crucial.

In conclusion, the combination of SERS and MNMs offers unprecedented opportunities for the in situ and real-time analysis of complex biological samples. These systems significantly enhance detection specificity, efficiency, sensitivity, and repeatability. The ongoing development of SERS-active MNMs will continue to unveil new possibilities, not only in biosensing and medical diagnostics but also in other domains such as environmental monitoring and industrial chemical analysis. With further advancements in material science, AI-driven data analysis, and fabrication technologies, SERS-active MNMs are well-positioned to become a cornerstone technology in the next generation of diagnostic and sensing platforms.

## Data Availability

Data sharing is not applicable to this article.
